# Tumour Budding in Oral Squamous Cell Carcinoma: A Narrative Review

**DOI:** 10.7759/cureus.69624

**Published:** 2024-09-17

**Authors:** Sonali Jakkulwar, Sunita Vagha, Minakshi Chaudhary

**Affiliations:** 1 Pathology, Jawaharlal Nehru Medical College, Datta Meghe Institute of Higher Education and Research, Wardha, IND; 2 Nursing, Shalinitai Meghe College of Nursing, Datta Meghe Institute of Higher Education and Research, Wardha, IND

**Keywords:** cancer progression, histopathology, oral squamous cell carcinoma, prognostic markers, tumour budding, tumour microenvironment

## Abstract

Tumour budding is an emerging prognostic factor in oral squamous cell carcinoma (OSCC) that reflects the invasive behaviour of the tumour. This narrative review aims to provide a comprehensive overview of the role of tumour budding in OSCC, synthesizing current research and clinical findings. We explore the definition and characterization of tumour budding, its correlation with histopathological features, and its impact on patient outcomes. Tumour budding is associated with increased local invasion, lymph node metastasis, and poor overall survival, highlighting its potential as a key marker for aggressive disease. This review also discusses the methods used to assess tumour budding, including histological scoring systems and the challenges in standardizing these assessments. By integrating findings from recent studies, we offer insights into the clinical relevance of tumour budding in OSCC management and propose future research directions to enhance its application in personalized treatment strategies.

## Introduction and background

Oral squamous cell carcinoma (OSCC) is a significant and prevalent malignancy within the realm of head and neck cancers, accounting for approximately 90% of oral cavity cancers [1]. It typically arises from the epithelial cells of the oral mucosa and presents with varying degrees of aggressiveness and clinical outcomes [2]. Key risk factors include tobacco use, alcohol consumption, and human papillomavirus (HPV) infection, which contribute to its pathogenesis [3]. Despite advances in diagnostic and therapeutic approaches, OSCC remains a significant cause of morbidity and mortality due to its tendency for late-stage diagnosis and high rate of local recurrence [4]. Tumour budding is a histopathological phenomenon characterized by small clusters or single cancer cells at the invasive front of a tumour [5]. This feature has gained attention in cancer research for its association with tumour aggressiveness, poor prognosis, and increased metastatic potential. In OSCC, tumour budding has been linked to adverse clinical outcomes, including higher rates of lymph node metastasis and reduced survival rates [6]. The evaluation of tumour budding can provide valuable prognostic information and help stratify patients who may benefit from more aggressive treatment approaches [7]. Thus, understanding the role of tumour budding in OSCC is crucial for enhancing prognostic accuracy and guiding therapeutic strategies.

This narrative review aims to comprehensively analyse the current research on tumour budding in OSCC. The primary objectives are to elucidate the role of tumour budding as a prognostic marker, examine its impact on patient outcomes, and assess its potential as a therapeutic target. The scope of this review includes a detailed evaluation of recent studies that investigate the correlation between tumour budding and clinical features of OSCC, as well as an exploration of the underlying biological mechanisms that contribute to its prognostic significance. By synthesizing current knowledge and identifying gaps in the literature, this review seeks to inform future research directions and improve the clinical management of OSCC.

## Review

Search methodology

This narrative review on "Tumour Budding in Oral Squamous Cell Carcinoma" used a comprehensive search strategy to identify relevant studies. Initially, a total of 142 studies were considered through systematic searches of electronic databases, including PubMed, Scopus, and Web of Science, using keywords such as "tumour budding," "oral squamous cell carcinoma," and "prognostic significance." After an initial screening of titles and abstracts, 86 studies were excluded based on predefined criteria, such as lack of focus on tumour budding, non-human studies, or irrelevance to oral squamous cell carcinoma. Further, after a full-text review, 23 studies were excluded due to methodological limitations, such as small sample sizes or lack of detailed reporting on tumour budding. Ultimately, 33 studies met the inclusion criteria and were comprehensively reviewed, providing a robust basis for the discussion of tumour budding's role and prognostic significance in oral squamous cell carcinoma.

Pathophysiology of oral squamous cell carcinoma

Cellular and Molecular Mechanisms of OSCC

*Oral squamous cell carcinoma* (OSCC): OSCC is a prevalent malignancy within the head and neck region, characterized by the uncontrolled proliferation of squamous epithelial cells in the oral cavity. Tumour budding, a histopathological phenomenon observed in OSCC, is marked by small clusters of cancer cells at the invasive front of the tumour. This process is indicative of a more aggressive phenotype and has been associated with poor prognosis, increased metastatic potential, and reduced overall survival rates. Tumour budding reflects the tumour's ability to invade surrounding tissues and can serve as a valuable biomarker for assessing the invasive and metastatic behaviour of OSCC. In this narrative review, we explore the significance of tumour budding in OSCC, its implications for disease progression, and its potential utility in guiding treatment strategies and improving patient outcomes [8].

*Epithelial-mesenchymal transition* (EMT): EMT plays a crucial role in tumour budding within OSCC, marking a significant shift in the behaviour and characteristics of cancer cells. EMT is a process wherein epithelial cells lose their cell-cell adhesion properties and gain mesenchymal features, including increased motility and invasiveness. A complex interplay of signalling pathways, transcription factors, and microenvironmental factors drives this transition. In OSCC, EMT facilitates the detachment of tumour cells from the primary mass, leading to the formation of small clusters or buds at the invasive front. These budding cells exhibit enhanced migratory and invasive capabilities, contributing to the progression of the disease and the potential for metastasis. Understanding the mechanisms of EMT in tumour budding can provide valuable insights into the aggressive nature of OSCC. It may inform the development of targeted therapies to mitigate tumour spread and improve patient outcomes [9].

*Wnt/β-catenin signalling pathway*: The Wnt/β-catenin signalling pathway plays a pivotal role in the development and progression of OSCC through its influence on tumour budding, a key indicator of aggressive cancer behaviour. In normal epithelial tissues, Wnt signalling regulates cell proliferation and differentiation by stabilizing β-catenin, which then translocates to the nucleus to activate target genes involved in these processes. However, in OSCC, aberrant activation of this pathway is frequently observed due to mutations in the Wnt pathway components or dysregulation of β-catenin degradation. This aberrant activation leads to the accumulation of β-catenin in the nucleus, promoting the expression of genes that drive the epithelial-to-mesenchymal transition, enhanced invasiveness, and tumour budding. The heightened tumour budding observed in OSCC is often associated with a worse prognosis and increased metastatic potential, underscoring the significance of the Wnt/β-catenin pathway in the aggressive phenotype of OSCC and highlighting it as a potential therapeutic target [10].

*Angiogenesis*: Angiogenesis plays a crucial role in tumour progression and budding as a pivotal mechanism supporting the tumour's growth and metastasis. Tumour budding, characterized by small clusters or single cancer cells at the invasive front of OSCC, is often associated with increased angiogenic activity within the tumour microenvironment. This enhanced angiogenesis facilitates the formation of new blood vessels, which are essential for providing the necessary nutrients and oxygen to sustain the expanding tumour mass. The interplay between tumour budding and angiogenesis underscores the complexity of OSCC pathogenesis, highlighting the need for targeted therapeutic strategies that can disrupt these processes and potentially improve patient outcomes. [11].

Role of Tumour Microenvironment in OSCC Progression

*The tumour microenvironment* (TME): The TME in OSCC plays a crucial role in the phenomenon of tumour budding, which is characterized by the presence of small clusters of tumour cells at the invasive front of the carcinoma. The TME in OSCC comprises a complex interplay of various cell types, including cancer-associated fibroblasts, immune cells, and endothelial cells, alongside an array of extracellular matrix components and signalling molecules. This microenvironment is often characterized by chronic inflammation, hypoxia, and altered matrix remodelling, which collectively contribute to the aggressive behaviour of the tumour and its ability to invade the surrounding tissues. The interactions between budding tumour cells and the TME not only facilitate local invasion but may also influence metastatic potential and resistance to therapy. Understanding these dynamic interactions within the TME is crucial for developing targeted therapeutic strategies to mitigate the adverse effects of tumour budding and improve patient outcomes in OSCC [12].

*Cancer-associated fibroblasts* (CAFs): CAFs play a crucial role in the progression and aggressiveness of oral squamous cell carcinoma (OSCC), particularly tumour budding. These fibroblasts, which are a key component of the tumour stroma, contribute to the creation of a pro-tumorigenic microenvironment that supports tumour budding. In this process, small clusters of cancer cells break away from the primary tumour and invade surrounding tissues. CAFs secrete various growth factors, cytokines, and extracellular matrix components that facilitate tumour cell migration and invasion. Their interactions with tumour cells enhance local invasion and promote resistance to therapy, making them a significant factor in tumour budding and overall tumour progression in OSCC. Understanding the precise mechanisms by which CAFs influence tumour budding can offer new insights into potential therapeutic targets for improving patient outcomes [13].

*Immune evasion*: It is another hallmark of OSCC, and the TME contributes to this by creating an immunosuppressive environment. Regulatory T cells (Tregs) and myeloid-derived suppressor cells (MDSCs) accumulate in the TME, inhibiting the activity of cytotoxic T cells and natural killer (NK) cells. This immune suppression allows tumour cells to evade immune surveillance and increase [14].

*Hypoxic environment*: The hypoxic environment within tumours plays a pivotal role in tumour budding, a process associated with aggressive tumour behaviour and poor prognosis. Hypoxia, or reduced oxygen availability, is commonly observed in solid tumours, including OSCC, due to inadequate vascularization and high metabolic demands. This low-oxygen state induces various cellular responses, contributing to tumour progression and metastasis. Hypoxia can promote EMT enhancing the invasiveness and stimulate the expression of various proangiogenic factors, all of which facilitate tumour budding. Tumour buds, which are small clusters of cells that invade surrounding stroma, are often found in hypoxic regions of OSCC, highlighting the interplay between the hypoxic microenvironment and the aggressive phenotypic traits of budding tumours. Understanding this relationship is crucial for developing targeted therapies aimed at mitigating the effects of hypoxia and improving outcomes for patients with OSCC [15].

Tumour Budding in OSCC

Tumour budding, defined as the presence of single cells or small clusters of cells at the invasive front of tumours, is a well-recognized phenomenon in OSCC and has been associated with poor prognosis. Tumour buds are thought to represent cells that have undergone EMT, allowing them to detach from the primary tumour mass and invade surrounding tissues. These buds interact with the TME, receiving signals that enhance their invasive capacity [5].

Concept of tumour budding

Definition and Characteristics of Tumour Budding

Tumour budding is defined as the presence of single cells or small clusters (less than five cells) of neoplastic epithelial cells at the invasive front of a carcinoma. It is a histopathological feature considered a hallmark of cancer invasion and metastasis, reflecting the tumour’s ability to disseminate from the primary site. In particular, tumour buds lack cell cohesion and exhibit a mesenchymal phenotype, often showing reduced expression of epithelial markers (e.g., E-cadherin) and increased expression of mesenchymal markers (e.g., vimentin), indicative of EMT [6]. This process plays a crucial role in the progression of many cancers, including OSCC, by facilitating local invasion and distant metastasis. Tumour budding is associated with poor prognosis, higher tumour grade, and increased risk of recurrence [16].

Historical Perspective on Tumour Budding in Cancer Research

The concept of tumour budding was first described in the context of colorectal cancer by Imai in 1954 when it was observed as “sprouting” cells at the invasive margins of tumours [17]. Since then, tumour budding has been extensively studied in various cancers, including OSCC, which is now recognized as an independent prognostic marker. Tumour budding was not widely accepted in the early years due to challenges in standardizing its assessment. Still, with advancements in histopathological techniques, including immunohistochemistry, the significance of tumour budding has gained greater recognition [18]. In OSCC, recent studies have highlighted the relevance of tumour budding in predicting clinical outcomes, leading to its inclusion in guidelines for tumour grading and treatment decision-making [17].

Tumour budding in oral squamous cell carcinoma

Epidemiology and Prevalence of Tumour Budding in OSCC

Tumour budding, a histopathological phenomenon defined by the presence of isolated or small clusters of cells (up to four or five) at the invasive front of carcinomas, has gained considerable attention in recent years as a prognostic marker in OSCC. Studies indicate that tumour budding is present in a significant proportion of OSCC cases, with rates ranging from 36% to 83%, depending on the criteria and cutoff points used for defining it. Its presence correlates with more aggressive tumour behaviour, including higher rates of lymph node metastasis, recurrence, and poor overall prognosis [19]. According to a meta-analysis by Almangush et al. (2024) [20], tumour budding is frequently seen in OSCC patients in the advanced stages and is associated with poor survival outcomes. The prevalence of high-grade tumour budding in OSCC is variable. Still, studies suggest that it occurs more commonly in advanced T stages of the disease, making it a crucial factor in tumour progression and metastasis [16]. The frequency of tumour budding also varies geographically and with lifestyle factors, such as tobacco and alcohol consumption, which are aetiological solid factors for OSCC [21].

Histological Features and Classification of Tumour Budding

Histologically, tumour budding is characterized by small clusters or single cancer cells seen at the invasive tumour front. These buds are typically identified using haematoxylin and eosin (H&E) staining. Still, immunohistochemical markers like cytokeratin may improve detection sensitivity, particularly in cases where buds are challenging to distinguish from the surrounding stromal cells. The assessment of tumour budding is now recognized by several guidelines, such as the International Tumour Budding Consensus Conference (ITBCC), which advocates for standardized reporting of tumour budding in routine practice [17]. Tumour budding is generally classified into low-grade and high-grade categories based on the number of buds present within a certain tumour area (most often 0.785 mm²). A high tumour budding score is associated with worse clinical outcomes, including lower disease-free and overall survival rates [22].

Comparison with Other Types of Cancers

While tumour budding is well-established in colorectal cancer, where it is recognized as an independent prognostic factor in staging and treatment planning, its relevance in OSCC has only recently begun to gain widespread recognition. In colorectal cancer, tumour budding is a strong predictor of lymph node metastasis, even in early-stage disease, and similar findings have been replicated in OSCC. Both cancer types show a clear correlation between high-grade TB and adverse outcomes [5]. In contrast, tumour budding in breast cancer is less commonly reported, though it shares histopathological characteristics with TB in OSCC and colorectal cancer. However, its prognostic utility is still under investigation. TB in lung adenocarcinoma and pancreatic ductal adenocarcinoma has also been explored, with findings showing that it plays a similar role in predicting poor outcomes in these cancers [12].

Clinical significance of tumour budding

Prognostic Implications of Tumour Budding in OSCC

Tumour budding, defined as the presence of isolated single cells or small clusters of up to five tumour cells at the invasive front of carcinomas, has emerged as a powerful prognostic marker in various malignancies, including OSCC. High-grade tumour budding is associated with an increased risk of recurrence, nodal metastasis, and poorer prognosis. Studies consistently show that higher tumour budding scores correlate with more aggressive disease phenotypes, making it a strong independent predictor of poor outcomes in OSCC [17].

Correlation with Clinical Outcomes (Survival Rates, Metastasis)

Tumour budding has been strongly linked to adverse clinical outcomes in OSCC. Several studies highlight the association between high tumour budding and reduced overall survival (OS) and disease-free survival (DFS). For instance, a meta-analysis reported that patients with high tumour budding have significantly lower five-year survival rates than those with low tumour budding [21]. Additionally, tumour budding has been correlated with an increased propensity for lymph node metastasis, a critical factor in determining prognosis and treatment decisions. In clinical practice, high tumour budding at the invasive front of OSCC is often a marker of more aggressive tumour behaviour, predicting a higher likelihood of local recurrence and distant metastasis [23].

Impact on Treatment Strategies and Patient Management

Given its significant prognostic value, tumour budding can influence treatment strategies and patient management in OSCC. Patients with high tumour budding may benefit from more aggressive surgical approaches, including wider excision margins and prophylactic neck dissection, even in early-stage disease. Additionally, the presence of high tumour budding may prompt the use of adjuvant therapies such as radiation or chemotherapy to reduce the risk of recurrence. By integrating tumour budding into standard histopathological evaluations, clinicians can better stratify patients into risk categories, allowing for personalized treatment plans that improve clinical outcomes [24].

Assessment and measurement of tumour budding

Methods for Evaluating Tumour Budding

Tumour budding, characterized by small clusters of cells at the invasive front of carcinomas, is a significant prognostic marker in various cancers, including OSCC [25]. The assessment of tumour budding involves primarily histopathological evaluation, typically conducted using H&E-stained sections. A high-power field (HPF) is often employed to count isolated single cells or small clusters of fewer than five cells. The number of buds per HPF or in a designated area of 0.785 mm², often called "hotspots," is quantified [26]. Several scoring systems are used to standardize the assessment of tumour budding. One of the most widely accepted is the ITBCC scoring system. This system categorizes tumour budding into three grades: low (0-4 buds), intermediate (5-9 buds), and high (≥10 buds) based on the count of buds in an HPF. Immunohistochemistry (IHC) staining, particularly for cytokeratins, is another valuable method in tumour buds that are difficult to distinguish from stromal cells due to poor differentiation [27].

Challenges and Limitations in Measurement

Despite the clinical relevance of tumour budding, its assessment in OSCC presents several challenges. One major issue is inter-observer variability. The subjective nature of manual counting under a microscope introduces variability in the number of buds identified by pathologists. This is further complicated by differences in tumour budding definitions across studies, with some pathologists counting only individual cells while others include clusters of cells [25]. Another limitation is the lack of a universal cutoff for what constitutes high tumour budding. Different studies may use varying thresholds, making it difficult to compare findings across different clinical settings and research studies [28]. Additionally, the method of evaluation (H&E staining vs IHC) can impact the visibility of tumour buds, with IHC offering more precise delineation in some cases, though at a higher cost and requiring more specialized resources [27]. Finally, technical challenges arise due to the heterogeneity of OSCC tissue. Tumour budding tends to occur at the invasive front, and uneven tissue processing or sectioning might result in missing relevant areas, thus underestimating the tumour bud count [2]. This emphasizes the need for standardized sampling techniques in assessing tumour budding.

Recent advances and research trends

Emerging Research on Tumour Budding and Its Molecular Markers

Recent advances in tumour budding have focused on identifying molecular markers that could provide deeper insights into its biological mechanisms and prognostic significance in OSCC. Emerging research highlights the importance of EMT as a key process involved in tumour budding. During EMT, epithelial cells lose their adhesion properties and acquire a more migratory and invasive phenotype, contributing to tumour budding. Molecular markers such as E-cadherin (an epithelial marker) and vimentin (a mesenchymal marker) have been widely studied in this context. A decreased E-cadherin and increased vimentin expression correlate with high tumour budding and poor prognosis in OSCC [29]. Moreover, recent studies have investigated the role of specific signalling pathways and gene mutations in tumour budding. For instance, the Wnt/β-catenin pathway has been implicated in regulating cell adhesion and migration, which are crucial in tumour budding. Mutations or dysregulation of this pathway may promote the detachment of cells from the primary tumour, facilitating budding. The expression of β-catenin, particularly its nuclear localization, has been associated with high tumour budding activity and a worse prognosis in OSCC [30]. Other molecular markers, such as the overexpression of matrix metalloproteinases (MMPs), have also been linked to the invasive potential of budding cells in OSCC [29].

Innovations in Diagnostic and Therapeutic Approaches Related to Tumour Budding

Diagnostic advances in evaluating tumour budding in OSCC have focused on improving the accuracy and reproducibility of assessment methods. Digital pathology and image analysis software has gained popularity in objectively quantifying tumour budding. These systems use algorithms to detect and count budding cells in scanned histopathological slides automatically, reducing observer variability and increasing the standardization of tumour budding assessment [31]. Furthermore, immunohistochemical (IHC) techniques that target cytokeratins or other epithelial markers have proven helpful in highlighting tumour buds, especially in cases where H&E staining alone makes it challenging to distinguish buds from stromal cells [32]. Therapeutically, research has begun to explore targeted treatments for patients with high tumour budding activity. Given the association between tumour budding and EMT, therapies targeting the EMT process have been investigated as a potential approach to suppress budding and limit tumour invasiveness. Inhibitors of the transforming growth factor-beta (TGF-β) signalling pathway, which plays a critical role in EMT, are currently being studied in preclinical models of OSCC with promising results. These inhibitors have been shown to reduce tumour cell migration and invasion, which could help control tumour budding and improve patient outcomes [33]. Additionally, novel approaches targeting molecular pathways involved in tumour budding, such as the Wnt/β-catenin signalling pathway, are being explored. In preclinical studies, small molecule inhibitors that block the activation of β-catenin have shown potential in reducing tumour growth and metastasis. These therapeutic innovations could pave the way for more personalized treatment strategies for patients with OSCC exhibiting high tumour budding activity [30].

Discussion

The narrative review reveals that tumour budding is a prognostic factor in OSCC. Tumour budding, characterized by small clusters of cells at the invasive front of tumours, is strongly associated with aggressive behaviour, including local invasion, metastasis, and poor overall survival [6]. Several studies have demonstrated that a higher tumour budding score correlates with a worse prognosis, particularly regarding lymph node metastasis and disease recurrence [23]. The ITBCC scoring system has emerged as the most widely used method to standardize the assessment, providing a framework to quantify budding in high-power fields and stratifying patients into low-, intermediate-, and high-risk categories [27]. IHC staining has further improved the accuracy of budding assessment, mainly when traditional H&E staining is insufficient to distinguish tumour cells from the surrounding stroma [16].

Implications for Clinical Practice and Future Research

Given its prognostic solid value, tumour budding should be integrated into routine pathological reports for OSCC. Incorporating tumour budding into existing staging systems could refine risk stratification and help tailor treatment plans more effectively. For instance, patients with high tumour budding may benefit from more aggressive treatment strategies, including adjuvant therapies such as radiotherapy or chemotherapy [25]. Additionally, using IHC for cytokeratin in budding assessment may enhance diagnostic precision, particularly in poorly differentiated tumours where the invasive front is challenging to delineate [27]. Future research should focus on establishing more standardized guidelines for tumour budding assessment in OSCC. While the ITBCC provides a useful framework, there is still variability in defining what constitutes a "bud" and what clinically significant thresholds across different regions and clinical settings [32]. Prospective studies are also needed to evaluate the predictive value of tumour budding in specific subgroups of OSCC patients, such as those with early-stage disease or different tumour locations. Additionally, research into the molecular mechanisms driving tumour budding could uncover potential therapeutic targets to inhibit this aggressive process.

Limitations of Current Research and Knowledge Gaps

Despite the growing evidence supporting tumour budding as a prognostic marker, several limitations remain. First, there is a lack of consensus on the precise cutoffs for low, intermediate, and high tumour budding. While the ITBCC provides some guidance, many studies use different thresholds, making it difficult to compare results across studies and integrate findings into clinical practice. Furthermore, most research has been retrospective, which introduces potential biases and limits the ability to establish causality [19]. Prospective, multi-centre studies are needed to confirm the prognostic significance of tumour budding and to establish universally accepted scoring systems. Another major limitation is the lack of standardization in the techniques used to assess tumour budding. Different staining methods, including H&E and IHC, can lead to discrepancies in bud counts. Although IHC is more sensitive, its higher cost and resource requirements may limit its widespread adoption, particularly in low-resource settings.

Additionally, the heterogeneous nature of OSCC means that tumour budding can vary widely even within a single tumour, and sampling errors can lead to an underestimation of its extent [27]. Finally, while tumour budding has been linked to worse outcomes in OSCC, the biological mechanisms driving this phenomenon are poorly understood. Studies exploring the molecular underpinnings of tumour budding, such as EMT and its relation to stem cell-like properties in OSCC, are still in their infancy. Addressing these knowledge gaps could pave the way for new therapeutic strategies targeting tumour budding.

## Conclusions

Tumour budding has emerged as a critical prognostic factor in OSCC, associated with higher rates of local invasion, lymph node metastasis, and poorer overall survival. Through histopathological evaluation, particularly with standardized scoring systems like the ITBCC, tumour budding can be quantified and used to stratify patients according to their risk of recurrence and prognosis. While histological assessment using H&E staining remains the most common approach, immunohistochemistry provides enhanced accuracy in cases of poorly differentiated tumours. However, challenges such as inter-observer variability, lack of universally accepted cutoff points, and technical limitations in tissue sampling and processing continue to impact the consistency of tumour budding assessment. Further research and international consensus are necessary to refine the measurement criteria and incorporate tumour budding into routine clinical practice. By addressing these limitations, tumour budding has the potential to become a more reliable tool for guiding treatment decisions in OSCC, ultimately improving patient outcomes.
